# Adult genitourinary sarcoma: analysis using hospital-based cancer registry data in Japan

**DOI:** 10.1186/s12885-024-11952-0

**Published:** 2024-02-15

**Authors:** Satoshi Nitta, Shuya Kandori, Kosuke Kojo, Shuhei Suzuki, Kazuki Hamada, Ichiro Chihara, Masanobu Shiga, Shotaro Sakka, Yoshiyuki Nagumo, Tomokazu Kimura, Bryan J. Mathis, Hiromitsu Negoro, Ayako Okuyama, Takahiro Higashi, Hiroyuki Nishiyama

**Affiliations:** 1 Department of Urology, Faculty of Medicine, University of Tsukuba, 1-1-1, Tennodai, 305-8575 Tsukuba, Ibaraki Japan; 2 International Medical Center, University of Tsukuba Affiliated Hospital, 2-1-1, Amakubo, 305-8576 Tsukuba, Ibaraki Japan; 3 Graduate School of Nursing, St Luke’s International University, 10-1, Akashi, Chuo-ku, 104-0044 Tokyo, Japan; 4 Institute for Cancer Control, National Cancer Center Japan, 5-1-1, Tsukiji, Chuo-ku, 104- 0045 Tokyo, Japan

**Keywords:** Genitourinary sarcoma, Registries, Survival, Urology

## Abstract

**Background:**

Genitourinary sarcomas are rare in adults and few large-scale studies on adult genitourinary sarcoma are reported. We aimed to elucidate the clinical characteristics, survival outcomes, and prognostic factors for overall survival of adult genitourinary sarcoma in Japan.

**Methods:**

A hospital-based cancer registry data in Japan was used to identify and enroll patients diagnosed with genitourinary sarcoma in 2013. The datasets were registered from 121 institutions.

**Results:**

A total of 116 men and 39 women were included, with a median age of 66 years. The most common primary site was the kidney in 47 patients, followed by the paratestis in 36 patients. The most common histological type was liposarcoma in 54 patients, followed by leiomyosarcoma in 25 patients. The 5-year overall survival rates were 57.6%. On univariate analysis, male gender, paratestis as primary organ, and histological subtype of liposarcoma were predictive of favorable survival while primary kidney, bladder, or prostate gland location were predictive of unfavorable survival. On multivariate analysis, primary paratestis was an independent predictor of favorable survival while primary kidney, bladder, or prostate gland were independent predictors of unfavorable survival.

**Conclusions:**

This is the first report showing the clinical characteristics and survival outcomes of adult genitourinary sarcoma in Japan using a real-world large cohort database.

**Supplementary Information:**

The online version contains supplementary material available at 10.1186/s12885-024-11952-0.

## Introduction

Soft tissue sarcomas represent less than 1% of all malignant tumors [1] while those originating in the genitourinary tract are exceedingly rare and represent approximately 2% of all soft tissue sarcoma and 1–2% of malignant genitourinary tumors [2, 3]. Genitourinary sarcomas are relatively common in children but uncommon in adults [2, 4, 5].

Few, relevant, large-scale studies on adult genitourinary sarcoma exist in the literature, with only 2 such studies (188 patients at the West China Hospital and 131 patients at the Memorial Sloan-Kettering Cancer Center) previously reported [3, 6]. While a recent analysis of adult genitourinary sarcoma based on the National Cancer Institute’s (NCI) Surveillance, Epidemiology, and End Results (SEER) registry was reported [1], the rarity of adult genitourinary sarcoma limits large-scale studies involving population-based cancer registry data. Therefore, it is important to clarify the clinical characteristics and survival outcomes of all genitourinary sarcomas in an adult population with whatever data is available.

In this study, our results are extrapolated from the Japanese hospital-based cancer registry (HBCR), which archives newly diagnosed cancer cases in designated cancer-care hospitals (DCCH) and other prefecture-recommended hospitals [7–9]. Using this database, we aimed to clarify the clinical characteristics, survival outcomes, and prognostic factors for overall survival of adult genitourinary sarcoma.

## Patients and methods

### Data source

HBCR data were submitted from registered institutions to the Center for Cancer Control and Information Services at the National Cancer Center in Japan. The data include patient age, sex, primary organ, histology, treatments, and survival outcome. We used HBCR data to identify all adult genitourinary sarcoma patients diagnosed in 2013 from 121 total institutions.

### Data extraction

**Fig. 1:**
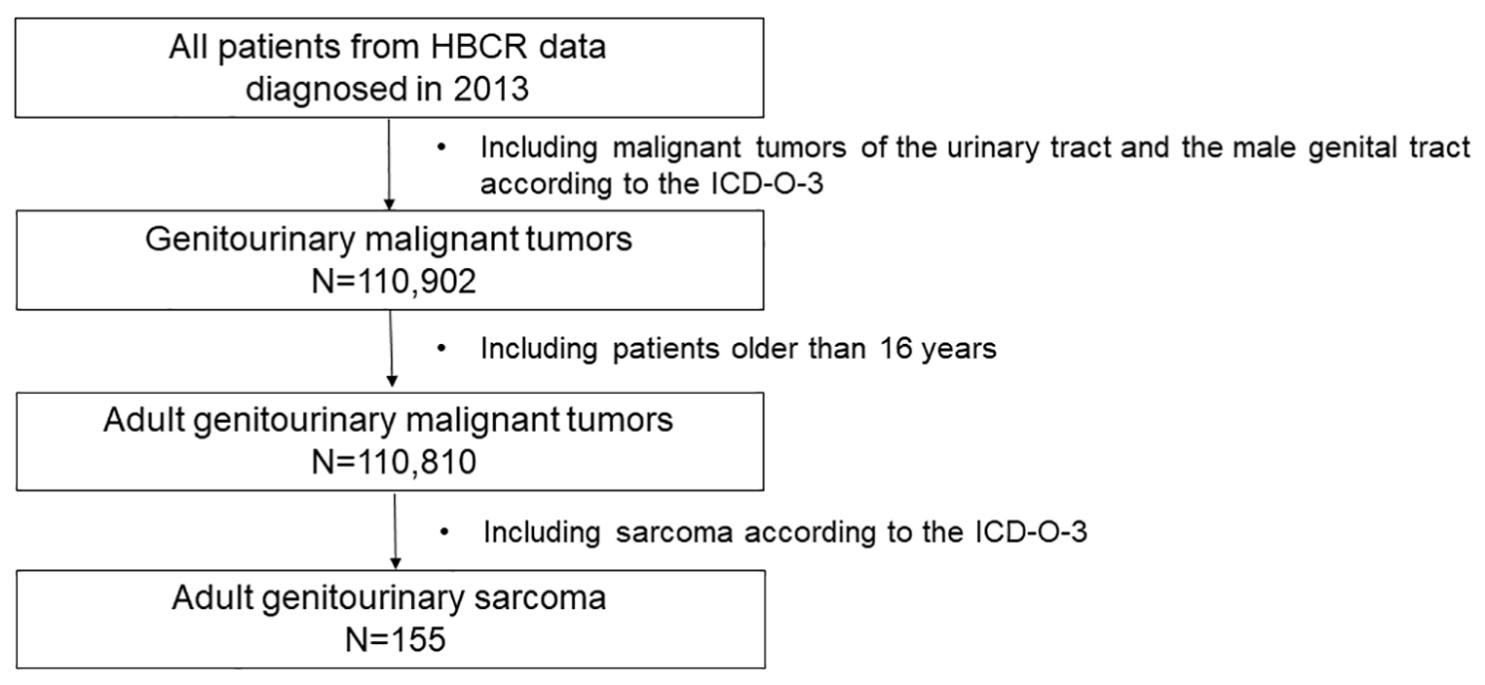
Eligibility of patients with adult genitourinary sarcoma

We identified patients diagnosed with adult genitourinary sarcoma from HBCR data by using the following criteria: patients who (1) were newly diagnosed with a malignant tumor of the urinary tract or the male genital tract using International Classification of Diseases for Oncology third edition (ICD-O-3) codes (Supplementary Table 1); (2) were diagnosed with genitourinary malignant tumor at ages older than 16 years; and (3) have a histologically confirmed sarcoma with ICD-O-3 histology codes (Supplementary Table 1).　According to previous studies, tumors of the female genital tract and retroperitoneum were excluded and an age cutoff was applied to form an adult cohort [3]. Finally, 155 patients with genitourinary sarcoma were included in this study (Fig. 1).

Primary organs were grouped as follows: kidney, bladder, prostate gland, testis,　paratestis, and others. Paratesticular tumors were defined as tumors that originated in the spermatic cord or epididymis [10]. Histological types were grouped as follows: liposarcoma, leiomyosarcoma, fibrous histiocytoma, rhabdomtosarcoma, carcinosarcoma, sarcoma not otherwise specified, and others.

### Statistical analysis

The overall survival (OS) time, which is the end-point in this study, was measured from the date of diagnosis to the date of death or last follow-up. Calculation of survival data was performed according to the Kaplan-Meier method and compared between groups by the log-rank test. Cox regression analysis was used to evaluate the prognostic factors of adult genitourinary sarcoma. The results of the Cox regression analysis are shown as hazard ratios (HR) with corresponding 95% confidence intervals (CI). P-values < 0.05 were considered statistically significant. GraphPad Prism8 (GraphPad Software, San Diego, CA) and SPSS® 25.0 for Windows® (SPSS Inc., Chicago, IL, USA) were used for the statistical analysis.

### Ethical considerations

This study protocol and data processing methods were approved by the Institutional Review Board of the University of Tsukuba (approval number: R03-228). All patients gave written, informed consent. All methods were performed in accordance with the Declaration of Helsinki and the Guideline of the University of Tsukuba. If a patient group was small (i.e. *n* < 10), we present only the approximate number to avoid identifying personal information, according to recommendations from the Ministry of Health, Labour and Welfare.

## Results

The search identified 110,810 patients with adult genitourinary tumors and, of these, 155 (0.1%) had adult genitourinary sarcoma. Table 1 summarizes patient characteristics. In 155 patients with genitourinary sarcoma, the most common primary organ was kidney in 47 patients (30.3%), followed by paratestis in 36 patients (23.2%), bladder in 19 patients (12.3%), testis in 18 patients (11.6%), and prostate gland in 12 patients (7.7%). The most common histological type was liposarcoma in 54 patients (34.8%), followed by leiomyosarcoma in 25 patients (16.1%). A total of 114 of 155 patients (73.5%) underwent surgery whereas 122 of 155 patients (78.7%) did not undergo chemotherapy. The number of patients who received radiation therapy was less than 10.

**Table 1:** Characteristics of 155 patients with adult genitourinary sarcoma

	Overall (*n* = 155)
Age	
Median	66
Range	19–88
Sex	
Male	116
Female	39
Primary organ	
Kidney	47
Bladder	19
Prostate gland	12
Testis	18
Paratestis	36
Others	23
Histology	
Liposarcoma	54
Leiomyosarcoma	25
Fibrous histiocytoma	< 10
Rhabdomyosarcoma	< 10
Carcinosarcoma	< 10
Sarcoma, not otherwise specified	15
Others	40
Treatment	
Surgery	114
Chemotherapy	33
Radiation therapy	< 10
Multimodal treatment	19

Supplementary Table 2 summarizes characteristics according to the primary affected organ. Stratified by primary organ, the most common histological subtypes in the kidney were leiomyosarcoma, followed by liposarcoma and sarcoma not otherwise specified. In the paratestis, the majority of histological subtypes were liposarcoma, followed by leiomyosarcoma and fibrous histiocytoma. In the bladder, leiomyosarcoma, carcinosarcoma, and sarcoma not otherwise specified were common. Stratified by histological subtype, liposarcoma was the most common in the paratestis, followed by the kidney and the testis, whereas leiomyosarcoma was most common in the kidney.

**Fig. 2:**
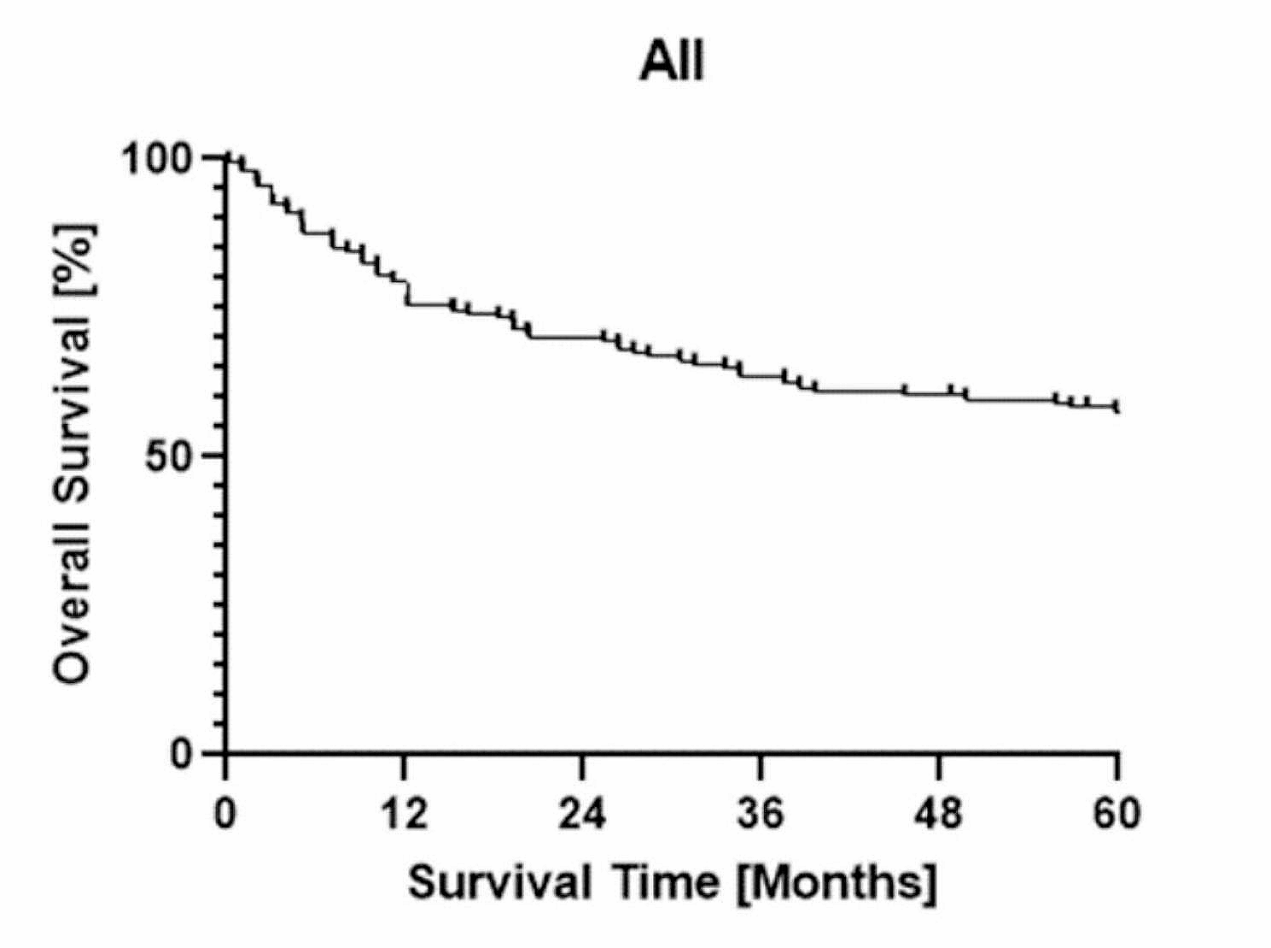
Overall survival in all patients with genitourinary sarcoma

**Fig. 3:**
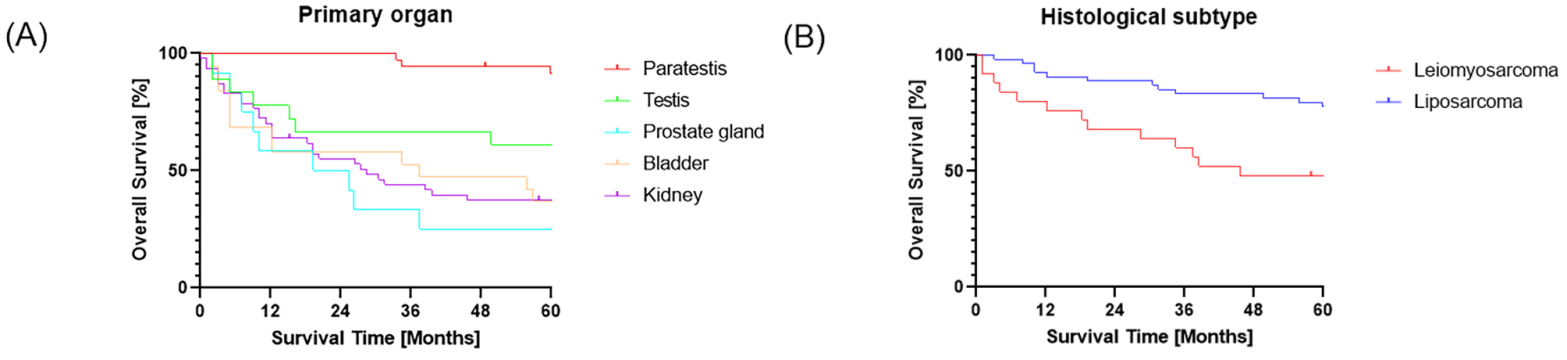
(**A**) Overall survival curves according to primary organ. (**B**) Overall survival curves according to histological subtype

We next analyzed OS rates. The median follow-up time was 67 months and the 5-year OS was 57.6% in patients with genitourinary sarcoma (Fig. 2). Stratified by primary organ, the 5-year OS for kidney, bladder, prostate gland, testis, and paratestis groups were 37.4%, 36.8%, 25.0%, 61.1%, and 91.6%, respectively (Fig. 3A). Stratified by histological type, the 5-year OS of liposarcoma and leiomyosarcoma groups were 77.8% and 48.0%, respectively (Fig. 3B).

We then used analytical techniques to determine significant predictors for the prognosis of genitourinary sarcoma. As shown in Table 2, univariate analysis showed that male gender (HR 0.498, 95% CI: 0.303–0.818, *p* = 0.006), primary kidney (HR 2.111, 95% CI: 1.309–3.406, *p* = 0.002), bladder (HR 2.185, 95% CI: 1.215–3.932, *p* = 0.009), prostate gland (HR 2.395, 95% CI: 1.185–4.838, *p* = 0.015) or paratestis (HR 0.136, 95% CI: 0.049–0.373, *p* < 0.001), histological type of liposarcoma (HR 0.342, 95% CI: 0.190–0.615, *p* < 0.001). Multivariate analysis showed that primary paratestis (HR 0.277, 95% CI: 0.091–0.846, *p* = 0.024) was an independent factor for favorable prognoses while primary kidney (HR 2.078, 95% CI: 1.009–4.280, *p* = 0.047), bladder (HR 2.319, 95% CI: 1.038–5.183, *p* = 0.040), or prostate gland (HR 2.636, 95% CI: 1.088–6.389, *p* = 0.032) were independent factors for unfavorable prognoses.

**Table 2:** Univariate and multivariate analysis of variables for overall survival of adult genitourinary sarcoma

	Univariate model				Multivariate model		
	HR	95% CI	p		HR	95% CI	p
Age (ref. <=60)							
> 60	1.294	0.781–2.144	0.316				
Sex (ref. Female)							
Male	0.498	0.303–0.818	**0.006**		1.035	0.578–1.852	0.909
Primary organ (ref. other organs)							
Kidney	2.111	1.309–3.406	**0.002**		2.078	1.009–4.280	**0.047**
Bladder	2.185	1.215–3.932	**0.009**		2.319	1.038–5.183	**0.040**
Prostate gland	2.395	1.185–4.838	**0.015**		2.636	1.088–6.389	**0.032**
Testis	0.847	0.388–1.850	0.677				
Paratestis	0.136	0.049–0.373	**0.000**		0.277	0.091–0.846	**0.024**
Histological subtype (ref. other histological subtypes)							
Liposarcoma	0.342	0.190–0.615	**0.000**		0.661	0.347–1.259	0.208
Leiomyosarcoma	1.262	0.691–2.306	0.449				

## Discussion

We analyzed a real-world cohort database to stratify adult genitourinary sarcomas by primary affected organ in Japan. In our series, 155 patients with genitourinary sarcoma were included based on an HBCR database and we noted the incidence and oncologic outcomes of genitourinary sarcoma.

First, we found that a majority of tumors were present in the kidney (30.3%), followed by the paratestis (23.2%) and bladder (12.3%). With the exception of the bladder as the most affected organ (27%), a study based on the SEER database similarly showed that the kidney (25%) and paratestis (17%) were commonly affected [1]. An analysis of 188 patients at a single institution in China showed a distribution by primary organ of the paratestis (30%), kidney (26%), and bladder (21%) [6]. Proportions vary, but our study is in line with others that report the paratestis, kidney, and bladder as primary sites for genitourinary sarcoma. These differences might be due to variances in population cohorts, but there has been no large-scale studies in Japan showing data comparable data to ours. Taken together, our data agrees with existing reports that these three organs are most likely to be affected.

The most commonly reported histological types of adult genitourinary sarcoma are liposarcoma and leiomyosarcoma [1, 3, 6, 11], similarly to our results where liposarcoma (34.8%) and leiomyosarcoma (16.1%) were the most prevalent. Furthermore, when stratified by primary organ, the observed proportion of liposarcoma was highest in the paratestis (66.7%), in contrast to leiomyosarcoma which was highest in the kidney (29.8%); these results are consistent with previous studies [1, 6].

Of note, the 5-year OS in our series was 57.6% and represents the first report on the prognosis of adult genitourinary sarcoma based on HBCR data in Japan. Previous reports in other small studies showed a 5-year OS for adult genitourinary sarcoma of 26–49% [2, 11, 12], in line with a study in China reporting a 5-year OS of 47.7% [6]. Our results, in contrast, demonstrated a higher 5-year OS outside of this reported range but, since we did not analyze stage, tumor grade, tumor size, and surgical margin, our results may not be directly comparable to the existing literature with regard to detailed clinical parameters. With regard to treatments, the proportion of patients without surgery was higher in our series compared to a report from a single institution in China (26.5% vs. 14.5%) [6]. Thus, it might be possible that our higher observed OS resulted from more appropriate application of surgery.

In our series, patients with paratesticular sarcoma had the highest OS compared with other primary organs. Multivariate analysis also showed that paratesticular sarcoma was associated with favorable prognoses in patients with adult genitourinary sarcoma. In previous reports, paratesticular sarcomas had relatively favorable prognoses with a reported 5-year OS of around 60–80%, which is superior to sarcoma originating in the kidney, bladder, or prostate gland [1, 2, 13–17]. The favorable prognosis may be explained by the anatomical site, which is easy to detect compared to the kidneys, bladder, or prostate gland [1]. The 5-year OS of paratesticular sarcoma in our series was superior to these previous reports. The majority histology type for paratesticular sarcoma in our study was liposarcoma, which in line with previous reports [1, 6, 18, 19]. However, while it was reported that the extent of differentiation was the most important factor affecting prognosis for patients with liposarcoma, a lack of available differentiation data in our study precludes direct comparisons in that regard [20].

By histological subtype, patients with liposarcoma had higher OS rates than those with leiomyosarcoma in our series. Nazemi et al. reported that 5-year OS rates of liposarcoma and leiomyosarcoma were respectively around 70% and 50%, based on the SEER database [1], which was comparable with ours. The histological subtype was a prognostic factor for overall survival on univariate analysis but not on multivariate analysis. This might be due to small sample size and a lack of liposarcoma differentiation data.

There are several limitations to this study. First, due to the retrospective and observational nature, we could not exclude the possibility of selection bias. Second, the study population was relatively small. Third, clinicopathological data, such as stage, tumor grade, tumor size, and surgical margin, were not available and the multivariate analysis could not be performed with these variables. The lacking of these variables makes comparative analyses with respect to previous studies arduous. Despite these limitations, the HBCR remains a trusted source of epidemiological data regarding rare adult genitourinary sarcoma in Japan. It is important to investigate biological findings to identify molecular biomarkers for incorporating into risk stratification and explore new targets for therapy. However, due to the rarity of adult genitourinary sarcoma, large-scale studies are currently limited. Further investigation into adult genitourinary sarcoma, including biological findings and therapeutic strategy, is thus required.

## Conclusions

Using a real-world large cohort database, we are the first to reveal that the kidneys are the primarily affected organs for adult genitourinary sarcoma in Japan. Additionally, paratesticular sarcoma had the most favorable outcomes compared with other primary organs.

### Electronic supplementary material

Below is the link to the electronic supplementary material.

Supplementary Material 1

## Data Availability

No datasets were generated or analysed during the current study.

## Abbreviations

CI: Confidence interval
DCCH: Designated cancer-care hospitals
HBCR: Hospital-based cancer registry
HR: Hazard ratio
ICD-O-3: International Classification of Diseases for Oncology third edition
NCI: National Cancer Institute
OS: Overall survival
SEER: The National Cancer Institute’s Surveilance, Epidemiology, and End Results
