# Supplementary material for: Adult genitourinary sarcoma: analysis using hospital-based cancer registry data in Japan
Source: BMC Cancer. 2024 Feb 15;24:215. doi: 10.1186/s12885-024-11952-0 (PMC10870526; doi:10.1186/s12885-024-11952-0)
Supplement: Supplementary file 1 — Supplementary Material 1 [file 12885_2024_11952_MOESM1_ESM.docx]

**Supplementary Table 1** **International Classification of Diseases for Oncology, 3rd edition (ICD-O-3) topographical and morphological codes included**

| **ICD-O-3 Topographical Codes Included** | **Primary site** |
| --- | --- |
| C60.1, C60.9 | Penis |
| C61.9 | Prostate gland |
| C62.0, C62.1, C62.9 | Testis |
| C63.0 | Epididymis |
| C63.1 | Spermatic cord |
| C63.2 | Scrotum |
| C63.8 | Male genital organs not otherwise specified |
| C64.9 | Kidney |
| C65.9 | Renal pelvis |
| C66.9 | Ureter |
| C67.0, C67.1, C67.2, C67.3, C67.4, C67.6, C67.7, C67.8, C67.9 | Urinary Bladder |
| C68.0 | Urethra |
| **ICD-O-3 Morphological Codes Included** | **Histology** |
| 8850, 8851, 8852, 8855, 8858 | Liposarcoma |
| 8890, 8891 | Leiomyosarcoma |
| 8830 | Fibrous histocytoma |
| 8960 | Nephroblastoma |
| 8964 | Clear cell sarcoma |
| 8810, 8811, 8813 | Fibrosarcoma |
| 8963 | Malignant rhabdoid tumor |
| 8802 | Giant cell sarcoma |
| 8935 | Stromal sarcoma |
| 9120 | Angiosarcoma |
| 9260 | Ewing sarcoma |
| 9180 | Osteosarcoma |
| 8982 | Malignant myoepithelioma |
| 9130 | Hemangioendothelial sarcoma |
| 9133 | Epithelioid hemangioendothelioma, malignant |
| 9364, 9473 | Primitive neuroectodermal tumor |
| 8900, 8910, 8912, 8920 | Rhabdomyosarcoma |
| 8800, 8805 | Sarcoma, NOS |
| 8815 | Solitary fibrous tumor |
| 8801 | Spindle cell sarcoma |
| 8980 | Carcinosarcoma, NOS |
| 8990 | Mesenchymal sarcoma |
| 9140 | Kaposi sarcoma |
| 9040, 9041, 9043 | Synovical sarcoma |

^†^ICD-O-3, International Classification of Diseases for Oncology, 3rd edition

**Supplementary Table 2 Patients characteristics according to tumor site**

|  | Kidney (N=47) | Bladder (N=19) | Prostate gland (N=12) | Testis  (N=18) | Paratestis (N=36) | Others (N=23) |
| --- | --- | --- | --- | --- | --- | --- |
| Age |  |  |  |  |  |  |
| Median | 60 | 74 | 55 | 72 | 67 | 71 |
| Range | 21-85 | 56-86 | 33-82 | 37-88 | 19-84 | 21-84 |
| Sex |  |  |  |  |  |  |
| Male | 23 | < 10 | 12 | 18 | 36 | 15-20 |
| Histology |  |  |  |  |  |  |
| Liposarcoma | 11 | < 10 | 0 | 11 | 24 | < 10 |
| Leiomyosarcoma | 14 | < 10 | < 10 | 0 | < 10 | < 10 |
| Fibrous histocytoma | < 10 | < 10 | 0 | 0 | < 10 | < 10 |
| Rhabdomyosarcoma | 0 | < 10 | < 10 | 0 | < 10 | < 10 |
| Carcinosarcoma | 0 | < 10 | 0 | 0 | 0 | < 10 |
| Sarcoma, not otherwise specified | < 10 | < 10 | < 10 | < 10 | 0 | < 10 |
| Others | 16 | < 10 | < 10 | < 10 | < 10 | < 10 |
| Treatment |  |  |  |  |  |  |
| Surgery | 28 | < 10 | < 10 | 18 | 35 | 19 |
| Chemotherapy | 16 | < 10 | < 10 | < 10 | < 10 | < 10 |
| Radiation therapy | < 10 | 0 | < 10 | < 10 | 0 | 0 |
| Multimodal treatment | < 10 | < 10 | < 10 | < 10 | < 10 | < 10 |
